# Perfusion weighted imaging in the assessment of the pathology and outcomes of lateral medullary infarction

**DOI:** 10.17712/nsj.2016.4.20160282

**Published:** 2016-10

**Authors:** Dao P. Zhang, Hong T. Zhang, Suo Yin, Fu L. Yan

**Affiliations:** *From the Department of Neurology (Zhang D, Zhang H), Department of Imageology (Yin), Zhengzhou People’s Hospital, Zhengzhou, and from the Department of Neurology (Yan), Zhongda Hospital affiliated Southeast University, Nanjing, China*

## Abstract

This series case report aimed to elucidate the underlying pathology and outcomes of lateral medullary infarction (LMI) using perfusion weighted imaging (PWI). Four patients were diagnosed with LMI based on high-field diffusion-weighted magnetic resonance imaging (MRI-DWI) and PWI. The national institutes of health stroke scale (NIHSS) scores were recorded on days 1, 7, and 30, and the Barthel index was assessed on days 7 and 30. Three patients exhibited relative regional hypoperfusion of medullary lesion in the perfusion maps. Two cases exhibited ipsilateral hypoperfusion in the inferior cerebellum, whereas one patient exhibited a relatively regional hyperperfusion in the medulla oblongata. The LMI patients with a high NIHSS score and low Barthel index on days 7 and 30 exhibited regional hypoperfusion. This report of 4 LMI cases provides preliminary evidence that regional hypoperfusion may contribute to worse outcomes in LMI.

Infarctions related to the medulla oblongata comprise complicated and variable clinical symptoms, such as vertigo, dysarthria, dysphagia, ataxia, hypesthesia, paralysis, and dyspnea, which depend on the affected medullary tissues and structures during infarction. Lateral medullary infarction (LMI) and medial medullary infarction (MMI) are defined according to the infarction location.^1^ The vertebral artery (VA) represents one of the most commonly affected arteries in LMI, and the incidence rate of vertebral artery hypoplasia (VAH) is approximately 10-15.6%.^2^,^3^ Increasing evidence suggests that VAH may represent a risk factor for posterior circulation stroke, especially posterior inferior cerebellar artery (PICA) and LMI.^4^ When VAH patients exhibit infarction risk factors, for example, hypertension, diabetes, smoking, or hypercholesteremia, the incidence of posterior circulation stroke increases.^5^ However, it remains unclear which vascular risk factors increase the incidence of LMI, as well as the specific outcomes involved in the small perforating artery or PICA lesion that result in LMI. VAH has been associated with a relative hypoperfusion in the dependent PICA territory even in the absence of manifest posterior circulation ischemia (PCI).^6^ To date, there have been a limited number of reports describing the clinical implications of perfusion weighted imaging (PWI) in cases of medullary infarctions. Few studies have focused on the clinical usefulness of PWI for the evaluation of stroke patterns or outcomes in LMI. In this report, 4 males LMI with VAH patients are presented who were characterized as heavy smokers and exhibited hyperhomocysteinemia, meanwhile, the outcomes and pathology by PWI were investigated.

## Case Report

### Case 1

**Figure 1:**
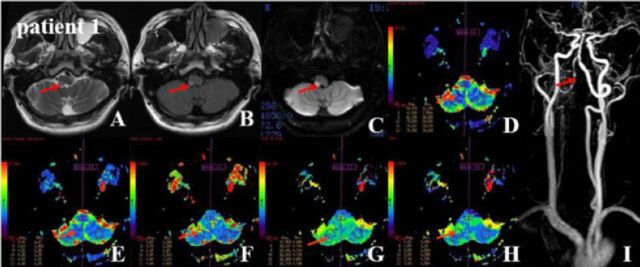
Magnetic resonance imaging-T2 weighted imaging, fluid attenuated inversion recovery (Flair) and diffusion weighted imaging showed **a-c)** high signal intensities in the right lateral medulla, **d-h)** Dynamic susceptibility contrast-enhanced-perfusion weighted imaging demonstrated a lesion ROI high cerebral blood flow, high cerebral blood flow, delayed mean transit time, decreased time to peak, and decreased time of maximum concentration on the VAH side. The representative rigid quantitative thresholds in the ROIs of 7 and 8 are shown in Table 2. **i)** constrast-enhanced megnetic resonance artery indicated right VAH and moderate stricture and rosary in the V4 segment of the right vertebral artery and minor kinking of the left vertebral artery. VAH - vertebral artery hypoplasia, ROI - region of interest

A 36-year-old male patient was hospitalized because of sudden vertigo and incompetency related to walking for 2 hours. The accompanying clinical symptoms included glossolalia, a sensation of spinning, nausea, and vomiting of stomach contents. He has an 18-year history of smoking 10-15 cigarettes per day and a 15-year history of drinking approximately 5 fluid ounces of liquor per day. He exhibited listlessness and dysarthria. He was positive for Horner’s sign (for example, ptosis, miosis, ipsilateral anhidrosis, and apparent enophthalmos) on the right side. Rotatory nystagmus was identified when the eyes moved towards the left side. He was unable to lift the right soft palate and exhibited a delayed pharyngeal reflex on the right side and abnormal results in the water swallow test (for example, he was asked to swallow 30 mL of warm water to judge the degree of dysphagia). He also exhibited an attenuated sensation of pain on the right side of the face and left side of the body, as well as inaccurate responses in the right side finger-nose test and heel-knee-tibia test (to determine ataxia). He exhibited right VAH with LMI. The head MRI diffusion weighted imaging (DWI), perfusion weighted imaging (PWI) and constrast-enhanced magnetic resonance artery (CEMRA) are shown in **Figure 1**. Blood test items (for example (e.g.), fasting blood glucose, cholesterol, homocysteine, or triglycerides) and major clinical characteristics (e.g., positive neurological signs, Barthel index, National Institutes of Health Stroke Scale, cerebral blood flow ratio, cerebral blood volume ratio) are shown in **Table 1**. Quantitative regional perfusion in the lateral medullary territory was shown in **Table 2**.

**Table 1:** General patient characteristics.

Variables	Case 1	Case 2	Case 3	Case 4
Age (years)	36	45	36	54
Gender	male	male	male	male
Hypertension history	-	-	-	+
Diabetes history	-	-	-	-
Coronary heart disease history	-	-	-	-
Smoking	+	+	+	+
Drinking	+	+	+	+
Homocysteine (umol/L)	50.88	15.57	39.75	16.14
Triglycerides (mmol/L)	1.37	1.81	3.17	3.64
Total cholesterol (mmol/L)	3.68	4.77	4.83	6.31
Low density lipoprotein (mmol/L)	1.94	3.55	3.46	4.01
Fasting blood glucose (mmol/L)	4.90	4.60	6.06	5.24
Glycosylated hemoglobin (%)	4.84	5.26	6.05	5.10
VAH diameter (mm)	0.9	1.7	1.9	0.4
LMI side	right	right	left	right
Horner’s sign	+	-	+	-
Dysarthria	+	+	+	-
Attenuated sensation	+	+	+	+
Limb paralysis	-	+	-	+
Ataxia	+	+	+	+
NIHSS day 1	4	8	4	6
NIHSS day 7	1	6	1	4
NIHSS day 30	1	4	1	4
Barthel index day 7	100	70	100	80
Barthel index day 30	100	80	100	90
CBF ratio	1.28	0.49	0.89	0.65
CBV ratio	1.29	0.69	0.89	0.56

VAH - vertebral artery hypoplasia; LMI - lateral medullary infarction, Horner’s sign - ptosis, miosis, ipsilateral anhidrosis, and apparent enophthalmos, NIHSS - National Institutes of Health Stroke Scale, CBF (cerebral blood flow)ratio - between the ischemic lesion and the contralateral mirror regions of interest, CBV (cerebral blood volum)ratio - between the ischemic lesion and the contralateral mirror regions of interest

**Table 2:** Quantitative regional perfusion in the lateral medullary territory of 4 cases.

Parameters of PWI	Case 1		Case 2		Case 3		Case 4	

	Left	Right	Left	Right	Left	Right	Left	Right
CBF	41.63±7.59	53.48±21.26	57.04±41.46	28.04±12.96	46.46±9.58	52.27±11.39	68.31±51.01	44.19±12.60
CBV	2.63±0.45	3.40±1.17	3.71±2.38	2.57±1.67	4.15±1.44	4.68±2.21	5.63±4.41	3.16±0.82
TTP	18.16±0.75	17.57±0.49	15.72±0.73	16.71±1.07	21.02±0.47	20.21±0.92	20.36±0.60	20.76±0.63
MTT	3.79±0.25	3.87±0.26	4.08±0.57	5.66±2.49	5.28±0.85	5.23±1.20	5.77±0.44	5.43±0.16
Tmax	4.50±0.00	4.32±0.49	3.30±0.71	4.30±1.08	4.67±0.47	4.73±0.54	4.31±0.50	4.50±0.00

CBF - cerebral blood flow, CBV - cerebral blood volume, TTP - time to peak, MTT - mean transit time, Tmax - time maximum, PWI - perfusion weighted imaging.

During hospitalization, he received antiplatelet agents, statins, scavenging free radicals, folic acid, mecobalamin, and vitamin B6. He was discharged at 7 days when they scored 100 on the Barthel index of daily life activities, which was used to assess the patients’ daily life activities, including 10 items (scores of 0-20 indicate very serious dysfunction; 20-45 indicate serious dysfunction; 50-70 indicate moderate dysfunction; 75-95 indicate mild dysfunction; and 100 indicates the individual can provide for oneself). He was no stroke relapse at the 3 month follow-up.

### Case 2

A 45-year-old male patient was hospitalized because of persistent dizziness and the inability to walk steadily for 2 days. The accompanying symptom included numbness on the left side of the body. He had a 20-year history of smoking approximately 20 cigarettes per day and a 25-year history of drinking approximately 8 ounces of liquor per day. He exhibited hemiparesis, facial palsy and an attenuated sensation of pain on the left side of the body. In addition, he was a dysarthria positive for Romberg sign (for example, when the patient was instructed to stand with the feet as close together as possible with his eyes closed, an abnormal result that comprised swaying or other evidence of instability was identified).

**Figure 2:**
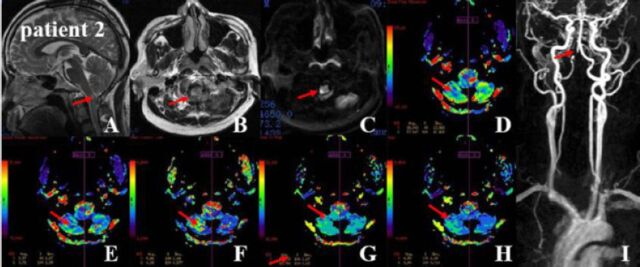
MRI-T2WI, Flair and DWI demonstrated **a-c)** high signal intensities in the right lateral medulla. **d-h)** Dynamic susceptibility contrast-enhanced-perfusion weighted imaging demonstrated a right ROI low CBF, low CBV, delayed TTP, delayed MTT, and delayed time maximum on the VAH side in Case 2. The representative rigid quantitative thresholds in the ROIs of 5 and 6 are shown in Table 2. **i)** constrast-enhanced megnetic resonance artery indicated right VAH and severe stricture and dilated region in the V4 segment of the right vertebral artery and moderate kinking of the left vertebral artery. VAH - vertebral artery hypoplasia, CBF - cerebral blood flow, CBV - cerebral blood volume, TTP - time to peak, MTT - mean transit time

The patient exhibited right VAH with LMI. The head MRI (DWI, PWI) and CEMRA are shown in **Figure 2**. Blood test items and major clinical characteristics are shown in **Table 1**. Quantitative regional perfusion in the lateral medullary territory was shown in **Table 2**. During hospitalization, the patient received antiplatelet agents, statins, scavenging free radicals, folic acid, mecobalamin, and vitamin B6. The patient was 80 on the Barthel index at 30 days. The patient was no stroke relapse at the 3 month follow-up.

### Case 3

**Figure 3:**
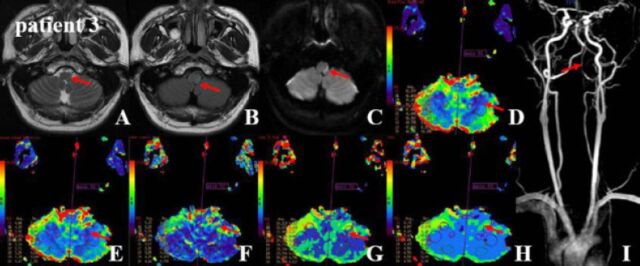
MRI-T2WI, Flair and DWI demonstrated **a-c)** high signal intensities in the left lateral medulla. **d-h)** Dynamic susceptibility contrast-enhanced-perfusion weighted imaging demonstrated left lesion ROI low CBF, low CBV, delayed MTT, and deceased Tmax on the VAH side. The representative rigid quantitative thresholds in the ROIs of 11 and 12 are shown in Table 2. **i)** Constrast-enhanced magnetic resonance artery indicated left VAH and minor stricture in the V4 segment of the left vertebral artery. VAH - vertebral artery hypoplasia, CBF - cerebral blood flow, CBV - cerebral blood volume, TTP - time to peak, MTT - mean transit time, ROI - region of interest

A 36-year-old male patient was hospitalized because of sudden vertigo and the inability to walk in a straight line (deviation to the left). The accompanying symptoms included hoarseness of the voice, difficulty swallowing, coughing while drinking water, low temperature on the left side of the face, heartache, and palmus. Vomiting approximately 150 ml of dark brown contents occurred on a single occasion. He had a 21-year history of smoking approximately 20 cigarettes per day and a history of occasional alcohol consumption. He exhibited dysarthria. He was positive for Horner’s sign on the left side of the body. Moreover, his nasolabial sulcus on the left side appeared to be shallow, and he exhibited tongue protrusion towards the left. He exhibited an attenuated sensation of pain on the right side of the body and inaccurate responses from the left side finger-nose test and heel-knee-tibia test. He also demonstrated a positive Romberg sign. He exhibited left VAH with LMI. The head MRI (DWI, PWI) and CEMRA are shown in **Figure 3**. Blood test items and major clinical characteristics are shown in **Table 1**. Quantitative regional perfusion in the lateral medullary territory was shown in **Table 2**. During hospitalization, he received antiplatelet agents, statins, scavenging free radicals, folic acid, mecobalamin, and vitamin B6. He was discharged at 7 days when they scored 100 on the Barthel index of daily life activities (scored 0-100). He was no stroke relapse at the 3 month follow-up.

### Case 4

**Figure 4:**
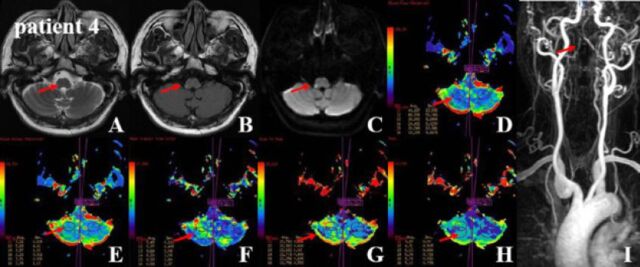
MRI-T2WI, Flair and DWI demonstrated **a-c)** high signal intensities in the right lateral medulla. **d-h)** DSC-PWI demonstrated the lesion ROI low CBF, low CBV, decreased TTP, delayed MTT, and delayed Tmax on the VAH side. The representative rigid quantitative thresholds in the ROIs of 11 and 12 are shown in Table 2. **i)** CEMRA indicated basilar artery hypoplasia, right vertebral artery hypoplasia, and severe kinking of the left vertebral V1 segment. VAH - vertebral artery hypoplasia, CBF - cerebral blood flow, CBV - cerebral blood volume, TTP - time to peak, MTT - mean transit time

A 54-year-old male patient was hospitalized because of incompetency of free movement on the left side of the body for 2 days. The accompanying symptoms included persistent dizziness and unsteady walking. He had a 35-year history of smoking approximately 20-30 cigarettes per day and a 30-year history of drinking approximately 5 ounces of liquor per day. He was diagnosed with hypertension with 160/110 mmHg, normal range=90-140/60-90 mmHg as the highest blood pressure level in the previous 2 years, but had not received regular treatment. Dyslipidemia and minor fatty liver were identified 2 months earlier; however, he did not follow up with regular treatment. He exhibited hemiparesis and an attenuated sensation of pain on the left side of the body and inaccurate responses in the right side finger-nose test and heel-knee-tibia test. He exhibited right VAH with LMI. The head MRI (DWI, PWI) and CEMRA are shown in **Figure 4**. Blood test items and major clinical characteristics are shown in **Table 1**. Quantitative regional perfusion in the lateral medullary territory was shown in **Table 2**. During hospitalization, he received antiplatelet agents, statins, scavenging free radicals, folic acid, mecobalamin, and vitamin B6. He was 90 on the Barthel index at 30 days. He was no stroke relapse at the 3 month follow-up. This study was approved by the Institutional Review Board of Zhengzhou People’s Hospital, Zhengzhou, China and all patients provided written informed consent.

## Discussion

Approximately 20-25% of all acute strokes occur in the posterior circulation. These strokes can be difficult to diagnose because they present in diverse ways and can easily be mistaken for benign entities. The present report indicated that male VAH patients who exhibited other vascular risk factors, such as smoking or hyperhomocysteinemia, had a LMI. These events were associated with the presence of an ipsilateral VAH on MRA. In the current case report, the patients exhibited infarction events and had more than three atherosclerotic or nonatherosclerotic risk factors for stroke. These findings raise the intriguing question as to whether VAH combined with multiple conventional vascular risk factors may be associated with LMI occurrence. Regional hypoperfusion is considered an important potential contributor to stroke risk in vertebrobasilar disease;^7^ however, the evaluation of hemodynamic status has traditionally been limited to the assessment of tissue perfusion in anterior circulation disease, with imaging techniques that poorly translate into the assessment of the more compact posterior circulation territory.^7^ In a recent study,^6^ VAH was associated with relative hypoperfusion in the dependent PICA territory in approximately ≤42% of patients without PCI. A previous case report described a symptom-free patient, 133Xe single photon emission computed tomography (SPECT) showed vertebrobasilar insufficiency because of an impaired vasodilatory response in both the occipital lobes and the right cerebellar hemisphere; the patient fell due to a severe ischemic infarction in these same areas past three weeks, which suggests that hypoperfusion may represent a valuable predictor of ischemia events. However, it was unclear whether perfusion predicted the outcomes and pathology of LMI.^8^ In the present cases, the results found that the LMI patients exhibited a relative regional hypoperfusion in the territory of the lateral medulla and inferior cerebellum, which suggests that the PICA trunk was involved, and hypoperfusion was associated with poor clinical outcomes. These findings supported the conclusion that PWI may represent a useful, novel prognostic tool for clinical outcomes following medullary infarctions. The series cases also showed that PWI exhibited a relative regional hypoperfusion only in the lateral medulla, which indicates that the small perforating artery was involved. Another remarkable finding was that PWI exhibited a relative hyperperfusion in the ipsilateral lateral medulla. It is well established that MRA is an accurate imaging modality for the assessment of vertebrobasilar system diseases. However, the use of only MRA results in limitations in the evaluation of VA branches. A lesion involved VA branches may display a PWI abnormality in the medulla or cerebellum without extremely concurrent occlusion or stenosis of the VA.

In summary, our cases extend previous findings and raise the intriguing question as to whether vascular risk factors and ipsilateral to VAH promote LMI events. Despite its technical limitations regarding spatial resolution and susceptibility to bone artifacts, especially in the posterior fossa, PWI is feasible to demonstrate perfusion abnormalities in acute LMI and may therefore facilitate an improved characterization of this stroke subtype with regard to its etiology and clinical course.
